# Comparison of Metabolism of Vitamins D_2_ and D_3_ in Children With Nutritional Rickets

**DOI:** 10.1002/jbmr.99

**Published:** 2010-04-01

**Authors:** Tom D Thacher, Philip R Fischer, Michael O Obadofin, Michael A Levine, Ravinder J Singh, John M Pettifor

**Affiliations:** 1 Department of Family Medicine, Mayo Clinic Rochester, MN, USA; 2 Department of Pediatric and Adolescent Medicine, Mayo Clinic Rochester, MN, USA; 3 Department of Family Medicine, Jos University Teaching Hospital Jos, Nigeria; 4 Division of Endocrinology and Diabetes, The Childrens Hospital of Philadelphia, and Department of Pediatrics, University of Pennsylvania School of Medicine Philadelphia, PA, USA; 5 Department of Laboratory Medicine and Pathology, Mayo Clinic Rochester, MN, USA; 6 MRC Mineral Metabolism Research Unit, Department of Paediatrics, University of the Witwatersrand Johannesburg, South Africa

**Keywords:** metabolic bone, vitamin D, calcium, pediatric, nutrition

## Abstract

Children with calcium-deficiency rickets may have increased vitamin D requirements and respond differently to vitamin D_2_ and vitamin D_3_. Our objective was to compare the metabolism of vitamins D_2_ and D_3_ in rachitic and control children. We administered an oral single dose of vitamin D_2_ or D_3_ of 1.25 mg to 49 Nigerian children—28 with active rickets and 21 healthy controls. The primary outcome measure was the incremental change in vitamin D metabolites. Baseline serum 25-hydroxyvitamin D [25(OH)D] concentrations ranged from 7 to 24 and 15 to 34 ng/mL in rachitic and control children, respectively (*p* < .001), whereas baseline 1,25-dihydroxyvitamin D [1,25(OH)_2_D] values (mean ± SD) were 224 ± 72 and 121 ± 34 pg/mL, respectively (*p* < .001), and baseline 24,25-dihydroxyvitamin D [24,25(OH)_2_D] values were 1.13 ± 0.59 and 4.03 ± 1.33 ng/mL, respectively (*p* < .001). The peak increment in 25(OH)D was on day 3 and was similar with vitamins D_2_ and D_3_ in children with rickets (29 ± 17 and 25 ± 11 ng/mL, respectively) and in control children (33 ± 13 and 31 ± 16 ng/mL, respectively). 1,25(OH)_2_D rose significantly (*p* < .001) and similarly (*p* = .18) on day 3 by 166 ± 80 and 209 ± 83 pg/mL after vitamin D_2_ and D_3_ administration, respectively, in children with rickets. By contrast, control children had no significant increase in 1,25(OH)_2_D (19 ± 28 and 16 ± 38 pg/mL after vitamin D_2_ and D_3_ administration, respectively). We conclude that in the short term, vitamins D_2_ and D_3_ similarly increase serum 25(OH)D concentrations in rachitic and healthy children. A marked increase in 1,25(OH)_2_D in response to vitamin D distinguishes children with putative dietary calcium-deficiency rickets from healthy children, consistent with increased vitamin D requirements in children with calcium-deficiency rickets. © 2010 American Society for Bone and Mineral Research.

## Introduction

Classically, vitamin D deficiency is distinguished from vitamin D insufficiency by the presence of marked biochemical perturbations in mineral metabolism and clinical and/or radiologic features of rickets or osteomalacia, although the severity of the signs and biochemical abnormalities do depend on the duration of the deficiency. Vitamin D–deficiency rickets is characterized by low circulating levels (typically <5 ng/mL) of 25-hydroxyvitamin D [25(OH)D], impaired intestinal calcium absorption, secondary hyperparathyroidism, and an exaggerated increase in serum 1,25-dihydroxyvitamin D [1,25(OH)_2_D] concentration after administration of vitamin D.(1–4) Although, worldwide, vitamin D deficiency is the principal cause of rickets, in some countries, such as Nigeria and Bangladesh, nutritional rickets results primarily from inadequate calcium intake.(5,6) Untreated Nigerian children with nutritional rickets have normal or only slightly reduced 25(OH)D values,(7) high fractional calcium absorption,(8) low dietary calcium intake,(7) markedly elevated 1,25(OH)_2_D concentrations, and a better response to treatment with calcium than with vitamin D.(9) These findings indicate that dietary calcium deficiency plays a more important role in the pathogenesis of rickets in Nigerian children than does vitamin D deficiency.

Operationally, vitamin D sufficiency represents a state in which the serum concentration of 1,25(OH)_2_D will not increase further after raising the concentration of 25(OH)D. Under these conditions, serum levels of substrate 25(OH)D are adequate to maintain the required 1,25(OH)_2_D concentrations.(10,11) Moreover, the concentration of 25(OH)D necessary to ensure normal mineral homeostasis will depend on the usual intake of dietary calcium. Not surprisingly, Nigerian children with rickets owing to dietary calcium deficiency have evidence for an increased requirement for vitamin D to optimize mineral metabolism.(12) Dietary calcium deficiency leads to secondary hyperparathyroidism, which decreases serum levels of 25(OH)D both directly, through increased conversion to 1,25(OH)_2_D, and indirectly, via enhanced degradation of 25(OH)D by the 24-hydroxylase enzyme CYP24A1, which is induced by 1,25(OH)_2_D.(13,14) Administration of vitamin D_2_ to Nigerian children with rickets produces a nearly twofold increase in 1,25(OH)_2_D concentrations, even though 1,25(OH)_2_D concentrations are markedly elevated prior to vitamin D administration. The increase in 1,25(OH)_2_D concentrations is similar to the response of children with vitamin D–deficiency rickets (although their baseline values are usually lower), consistent with the presence of secondary hyperparathyroidism and suggesting that vitamin D requirements are increased in calcium-deficiency rickets.(12) Consequently, we hypothesize that vitamin D requirements are increased by dietary calcium deficiency, but whether the low vitamin D status contributes to the pathogenesis of rickets in Nigerian children is currently unknown.

Vitamin D_3_ is the endogenous form of vitamin D produced by keratinocytes in the skin in response to ultraviolet B radiation from sunlight. Vitamin D_2_ is produced by irradiation of plant ergosterol and is used commonly for supplementation and food fortification. Both forms of vitamin D are hydroxylated in the liver to 25(OH)D. It has been suggested that vitamin D_3_ may be superior to vitamin D_2_ in sustaining adequate 25(OH)D values in adults(15,16) because 25(OH)D_2_ may bind less avidly to vitamin D–binding protein and be cleared more rapidly than 25(OH)D_3_. However, others have found that regular supplementation with both forms of vitamin D were equally effective in maintaining 25(OH)D levels.(17,18) The aim of this study was to compare the short-term metabolism of vitamin D_2_ with that of vitamin D_3_ in children with rickets and in healthy control children without rickets.

## Materials and Methods

We recruited children with genu varus or valgus deformities characteristic of rickets from the outpatient department of the Jos University Teaching Hospital in Nigeria. Radiographs of the wrists and knees were obtained. Those with active rickets, defined as a radiographic score of 1.5 or greater on a 10-point scale of severity,(19) were invited to participate in the study. Approval for the study was obtained from the Ethical Committee of the Jos University Teaching Hospital and the Institutional Review Board of the Mayo Clinic, and written informed consent was obtained from a parent of each enrolled child.

Healthy control children were recruited by asking each parent of a child with rickets to invite a child without clinical evidence of rickets of the same age and gender, who was a neighbor or relative, to participate. In cases where a neighbor or relative control was unable to be recruited, control children were recruited among the staff of the outpatient department. Transportation costs were paid for all enrolled children, and a gift valued at approximately $10 was provided to each enrolled child. Children with rickets were provided with 6 months of treatment with calcium and vitamin D at no cost after completing the 2-week study. Calcium was withheld during the study to avoid confounding the relationships between biochemical variables, allowing all changes to be attributed to the effect of vitamin D.

Data were collected from all children regarding usual sunlight exposure and the frequency and quantity of dairy product intake. The percentage of unclothed skin at enrollment was estimated by using a Lund and Browder age-related burn chart. Control children were randomly assigned by lottery to receive either vitamin D_3_ (cholecalciferol; Bio-Tech, Fayetteville, AR, USA) or vitamin D_2_ (ergocalciferol; Pliva, Inc., East Hanover, NJ, USA) as a single oral dose of 1.25 mg (50,000 IU). Although the potency of each form of vitamin D was identical, the matrix materials were not identical for the two products. Venous blood was collected at baseline and at 1, 3, 7, and 14 days after administration of vitamin D. All rachitic children in this study were given a single dose of vitamin D_3_ because the data on the metabolism of vitamin D_2_ in rickets were obtained from a previous study of 16 Nigerian children with rickets using vitamin D_2_ with an identical study protocol and laboratory.(12)

Serum samples were transported frozen to the Mayo Clinic for analysis. Serum calcium, phosphorus, alkaline phosphatase, and albumin were measured by standard methods. We measured all vitamin D metabolites by isotope-dilution liquid chromatography–tandem mass spectrometry (LC-MS/MS).(20) Unless otherwise indicated by subscript notation for individual metabolites, 25(OH)D, 24,25(OH)_2_D, and 1,25(OH)_2_D refer to the total concentrations of the D_2_ and D_3_ forms of each metabolite.

In the subgroup of children with rickets in the previous study that received vitamin D_2_, 25(OH)D and 1,25(OH)_2_D concentrations had been determined in the same laboratory by RIA (DiaSorin, Stillwater, MN, USA). Stored sera were available for 33 of the original 80 samples from children with rickets who had been given vitamin D_2_ and had vitamin D metabolites measured by RIA. In order to assess the agreement between values obtained by RIA and MS, we measured 25(OH)D and 1,25(OH)_2_D concentrations in these 33 samples with the MS methodology used in this study. Values obtained by MS and RIA were highly correlated for both 25(OH)D (*r* = 0.90, slope = 0.94 for MS as a function of RIA with intercept set at 0) and 1,25(OH)_2_D (*r* = 0.92, slope = 1.35), indicating that both methods gave similar values.

Data analysis and anthropometric calculations were performed with Epi Info 3.5.1 (CDC, Atlanta, GA, USA) and Excel 2003 (Microsoft Corp., Redmond, WA, USA). Nutritional anthropometric variables were calculated with the Nutrition program of Epi Info with Centers for Disease Control and Prevention (CDC)/World Health Organization (WHO) 1978 reference growth curves. A paired *t* test was used to compare values of continuous normally distributed variables with baseline values, and an unpaired *t* test was used to compare values of continuous variables between groups. A linear regression analysis was used to assess the difference in 25(OH)D concentrations at days 7 and 14 between the vitamin D_2_ and vitamin D_3_ groups while controlling for peak 25(OH)D concentration on day 3 as a covariate. *p* values less than .05 were considered significant.

## Results

A total of 46 children with lower extremity deformities were screened, and 13 children (4 males, 9 females) with radiographic evidence of rickets were recruited to receive vitamin D_3_. One child with rickets who received vitamin D_3_ was excluded from analysis because her response was an extreme outlier (described below). A total of 23 control children were recruited. One control child who received vitamin D_3_ did not return after the baseline visit and was excluded from the analysis. A second control child who failed to demonstrate an increase in serum concentration of 25(OH)D after administration of vitamin D_2_ was excluded from analysis because of doubts that he actually ingested the dose of vitamin D_2_. Data from 16 previously studied children with active rickets who had received vitamin D_2_ using the same protocol were included in the analysis.

Baseline characteristics of enrolled children are shown in Table 1. Baseline serum levels of 25(OH)D, 1,25(OH)_2_D, and 24,25(OH)_2_D differed significantly between rachitic and control children (*p* < 0.001). Among rachitic children, age, radiographic scores, and most biochemical values differed significantly (*p* < .01) between the vitamin D_2_ and vitamin D_3_ groups, consistent with more severe rickets in the group that received vitamin D_2_. However, the serum calcium concentration was lower in the vitamin D_3_ group than in the vitamin D_2_ group (*p* = .003). None of the children with rickets had severe vitamin D deficiency [serum concentration of 25(OH)D < 5 ng/ml]. Owing to their lower extremity deformities, children with rickets had reduced height for age, but they did not differ in weight for height from control children. Calcium intakes derived from dairy products were low in all groups and significantly lower in children with active rickets than in control children (*p* = .002).

**Table 1:** Baseline Characteristics of Nigerian Children With Nutritional Rickets and Healthy Control Children^a^

	Rachitic children		Control children		
			
Characteristic	Vitamin D_2_ (*n* = 16)	Vitamin D_3_ (*n* = 12)	Vitamin D_2_ (*n* = 11)	Vitamin D_3_ (*n* = 10)	Reference range
Age (months)	31 (15–48)^b^	71 (23–120)	39 (22–57)	31 (19–59)	
Sex (M:F)	9:7	4:8	5:6	5:5	
Duration of symptoms (months)	14 (0.1–34)^b^	41 (5–102)			
Radiographic score	4.0 (2.0–10.0)^b^	1.6 (1.5–7.0)			0 (normal XR)
Exposure to sunlight (h/day)	0.9 (0–12)	3.8 (0.5–5)	5.0 (0.5–10)	5.0 (2–6)	
Exposed skin (%)	47 (43–57)	50 (43–57)	47 (43–57)	43 (43–53)	
Dairy product calcium intake (mg/day)	31 (0–71)	15 (0–271)	71 (0–522)	115 (0–613)	
Height for age *Z*-score	−3.4 (−5.7 to −1.5)	−3.8 (−5.5 to −0.3)	0.7 (−0.9 to 2.9)	−0.4 (−1.9 to 2.3)	−2.0 to 2.0
Weight for height *Z-*score	−0.7 (−2.7 to 1.9)^b^	0.4 (−1.6 to 1.9)	−0.4 (−2.0 to 0.9)	−0.2 (−2.0 to 1.0)	−2.0 to 2.0
Serum biochemistry					
Calcium (mg/dL)^c^	9.4 (7.7–10.0)^b^	7.9 (6.1–9.1)	9.3 (8.7–10.3)	9.8 (8.6–10.4)	9.6–10.6
Phosphorus (mg/dL)^d^	3.5 (2.1–4.4)	4.1 (2.8–6.6)	4.7 (4.2–5.5)	5.1 (4.4–6.5)	3.7–5.4
Alkaline phosphatase (U/L)	732 (451–1268)^b^	412 (275–1552)	167 (89–196)	178 (105–230)	149–476
Albumin (g/L)	43 (39–46)	39 (31–45)	43 (38–47)	42 (35–46)	35–50
25(OH)D (ng/mL)^e^	11 (7–16)^b^	15 (11–24)	26 (21–34)	28 (15–33)	25–80^f^
1,25(OH)_2_D (pg/mL)^g^	175 (120–330)^b^	271 (166–390)	101 (90–178)	125 (72–208)	24–86
24,25(OH)_2_D (ng/mL)^h^	0.98^i^ (0.33–1.7)	0.90 (0.43–2.8)	4.11 (2.39–5.84)	4.27 (1.44–5.94)	Not established
24,25(OH)_2_D/25(OH)D proportion (%)	10 (5–13)	7 (4–16)	16 (9–23)	14 (10–33)	Not established

a Data are shown as median values (range).

b *p* < .01 for comparison with the vitamin D_3_ group.

c To convert values for calcium to millimoles per liter, multiply by 0.25.

d To convert values for phosphorus to millimoles per liter, multiply by 0.32.

e To convert values for 25(OH)D to nanomoles per liter, multiply by 2.50.

f Optimal range.

g To convert values for 1,25(OH)_2_D to picomoles per liter, multiply by 2.40.

h To convert values for 24,25(OH)_2_D to nanomoles per liter, multiply by 2.40.

i *n* = 10.

Serum levels of vitamins D_2_ and D_3_ were measured in control children, and neither was detectable at baseline. One day after vitamin D administration, serum vitamin D_2_ was 88.4 ± 27.2 ng/mL in the vitamin D_2_ group, and serum vitamin D_3_ was 148.9 ± 47.6 ng/mL in the vitamin D_3_ group of control children (*p* = .002).

**Fig. 1:**
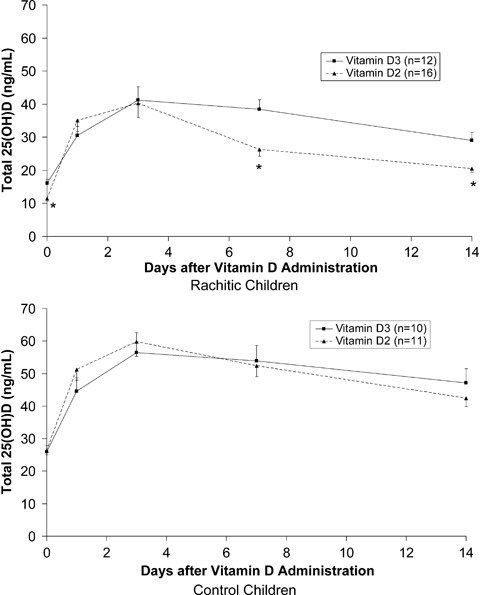
Mean serum concentrations of 25(OH)D in response to oral vitamin D_2_ or vitamin D_3_ administration (1.25 mg) in children with nutritional rickets and control children. Bars indicate standard error of the mean, and asterisks denote *p* < .01 for comparison with vitamin D_3_ group.

Total 25(OH)D concentrations peaked on day 3 (Fig. 1), and incremental changes were similar in the rachitic children who received vitamin D_2_ or vitamin D_3_ (29 ± 17 and 25 ± 11 ng/mL, respectively) and in control children after vitamin D_2_ or vitamin D_3_ administration (33 ± 13 and 31 ± 16 ng/mL, respectively). These data suggest that the bioavailability of both forms of vitamin D was similar despite differences in the matrices of the two preparations and greater levels of serum vitamin D_2_ on day 1. The day 3 concentration of total 25(OH)D was less than 30 ng/mL in 6 (21%) of the children with rickets and in none of the control children. The highest values of 25(OH)D, attained on day 3, were 77 and 101 ng/mL in rachitic and control children, respectively. The disappearance rate of total 25(OH)D after day 3 was similar after vitamin D_2_ and vitamin D_3_ administration in control children, suggesting that under conditions of normal mineral homeostasis, these two forms of vitamin D are bioequivalent. In a linear regression analysis controlling for the 25(OH)D concentration on day 3 as a covariate, there was a greater decline in 25(OH)D concentrations on day 7 (*p* < .001) and on day 14 (*p* < .001) in the vitamin D_2_ group than the vitamin D_3_ group among children with rickets, consistent with a more rapid clearance of 25(OH)D_2_ than 25(OH)D_3_. Although the group that received vitamin D_2_ had lower baseline 25(OH)D values than the vitamin D_3_ group, both groups achieved similar peak levels of 25(OH)D on day 3.

**Fig. 2:**
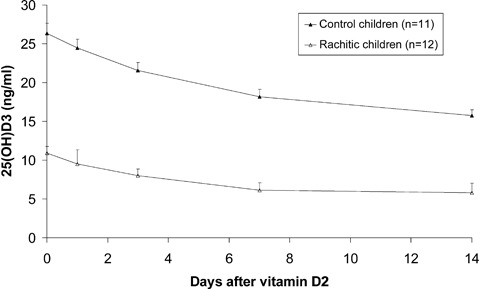
Change in serum 25(OH)D_3_ after administration of oral vitamin D_2_ administration in healthy control children and in children with rickets. Bars indicate standard error of the mean.

Figure 2 depicts the decline in endogenous 25(OH)D_3_ concentrations following the administration of vitamin D_2_ to rachitic and control children. Endogenous 25(OH)D_3_ concentrations declined gradually in both rachitic and control children, reaching 53% and 59% of baseline values by day 14.

**Fig. 3:**
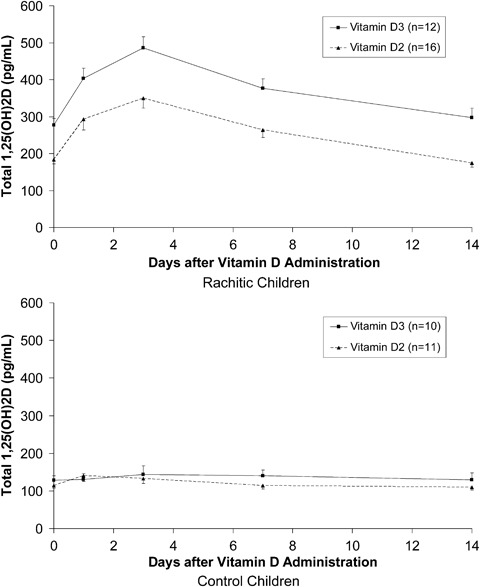
Mean serum concentrations of 1,25(OH)_2_D in response to oral vitamin D_2_ or vitamin D_3_ administration in children with nutritional rickets and control children. Bars indicate standard error of the mean.

Figure 3 shows the response of 1,25(OH)_2_D concentrations to vitamin D administration. The significant increase (*p* < .001) in 1,25(OH)_2_D concentrations from baseline to day 3 was similar for both forms of vitamin D in rachitic children (166 ± 80 and 209 ± 83 pg/mL after vitamin D_2_ and vitamin D_3_ administration, respectively; *p* = .18). In the children with rickets, 1,25(OH)_2_D concentrations nearly doubled in response to both vitamin D_2_ and vitamin D_3_. Moreover, in the two groups of children with rickets, the rates of decline of 1,25(OH)_2_D values after day 3 were similar. In contrast to the rachitic children, control children did not increase their 1,25(OH)_2_D concentrations in response to vitamin D administration.

One child with rickets who received vitamin D_3_ was excluded from analysis as an outlier because her response clearly differed from all other children with rickets. She had a relatively low baseline 1,25(OH)_2_D concentration (77 pg/mL) and had no change in 1,25(OH)_2_D (71 pg/mL on day 3) despite an increase in 25(OH)D concentration from 32 to 69 ng/mL after administration of vitamin D_3_. Her biochemical profile was consistent with hypophosphatemic rickets.

**Fig. 4:**
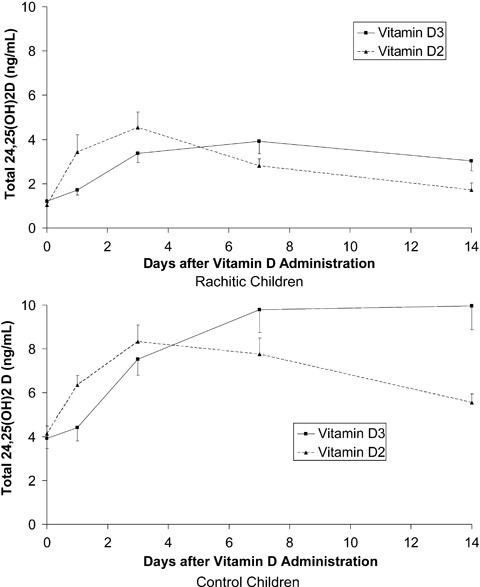
Mean serum concentrations of 24,25(OH)_2_D in response to oral vitamin D_2_ or vitamin D_3_ administration in children with nutritional rickets and control children. Bars indicate standard error of the mean.

**Fig. 5:**
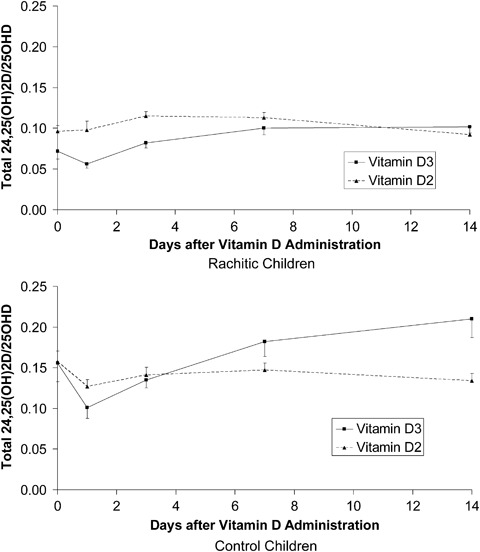
Mean 24,25(OH)_2_D concentrations as a proportion of 25(OH)D concentrations in response to oral vitamin D_2_ or vitamin D_3_ administration in children with nutritional rickets and control children. Bars indicate standard error of the mean.

Serum 24,25(OH)_2_D showed an initial increase after vitamin D administration (Fig. 4). In both rachitic and control children, values of 24,25(OH)_2_D rose more rapidly, peaking earlier (day 3 compared with day 7) and declining more rapidly after administration of vitamin D_2_ than after vitamin D_3_, suggesting more rapid catabolism of 25(OH)D_2_ than 25(OH)D_3_ and a more rapid clearance of 24,25(OH)_2_D_2_. The incremental areas under the curve for 24,25(OH)_2_D were 26.1 ± 13.0 and 28.6 ± 18.6 ng/mL per day in rachitic children who received vitamins D_2_ and D_3_, respectively (*p* = .03). In control children, the incremental areas under the curve for 24,25(OH)_2_D (41.0 ± 20.6 and 65.0 ± 27.8 ng/mL per day for the vitamin D_2_ and vitamin D_3_ groups, respectively; *p* = .04) were significantly greater than those of rachitic children (*p* = .002). In both groups of children with rickets, total 24,25(OH)_2_D was highly correlated with total 25(OH)D at all time points (*r* = 0.84 to 0.96). In the two groups of control children, the relationship also was positive, but the correlation was less (*r* = 0.64 to 0.76). Figure 5 displays the 24,25(OH)_2_D concentrations as a proportion of 25(OH)D concentrations, which were significantly greater at baseline in control children (16% ± 6%) than in rachitic children (6% ± 3%, *p* < .001), suggesting that the rachitic children converted a greater proportion of 25(OH)D to 1,25(OH)_2_D than to 24,25(OH)_2_D prior to administration of vitamin D. After vitamin D administration, all groups of children demonstrated an initial fall in the proportion of 24,25(OH)_2_D on day 1 owing to the rapid increase in 25(OH)D concentrations.

The increment in 1,25(OH)_2_D was unrelated to baseline 25(OH)D concentrations (*r* = −0.22, *p* = .24). Unlike our previous study, in which we demonstrated a positive relationship between 1,25(OH)_2_D and 25(OH)D in children with rickets after administration of vitamin D_2_, we found no significant correlations at any time point between 1,25(OH)_2_D and 25(OH)D in children with rickets after administration of vitamin D_3_. Similarly in the control children, no significant relationships between 1,25(OH)_2_D and 25(OH)D were found after administration of either vitamin D_2_ or vitamin D_3_.

## Discussion

In the short term, vitamins D_2_ and D_3_ appear to produce similar increases in 25(OH)D concentrations in both rachitic and healthy children. The increment in 25(OH)D in response to a single oral dose of 1.25 mg of vitamin D_2_ or vitamin D_3_ was approximately 30 ng/mL (approximately 0.6 ng/1000 IU of vitamin D) both in children with rickets and in healthy control children. The peak concentration of 25(OH)D that occurred 3 days after vitamin D administration is earlier than that seen in adults (7 days).(21,22) We surmise that this may be related to a greater activity of hepatic 25-hydroxylase in children compared with adults. Indirect evidence for this is the observation that older adults have a less rapid rise and a slower clearance of 25(OH)D than younger adults.(21)

The serum concentration of 25(OH)D_3_ declined gradually after administration of oral gradually D_2_ in both healthy control children (33% reduction by day 14) and children with rickets (50% reduction by day 14). In the children with rickets, this effect could be due to both induction of CYP24 by increasing concentrations of 1,25(OH)_2_D and competitive displacement of 25(OH)D_3_ from vitamin D–binding protein by 25(OH)D_2_. Because there was no significant increase in circulating 1,25(OH)_2_D after administration of vitamin D_2_ to normal children, the decline in serum 25(OH)D_3_ in these children was likely due solely to displacement from vitamin D–binding protein. We saw no difference between the disappearance rate of 25(OH)D after administration of vitamin D_2_ or vitamin D_3_ in the control groups, which suggests that these two forms of vitamin D are bioequivalent in normal healthy children. By contrast, in children with rickets, there was a suggestion of a more rapid decline in 25(OH)D after vitamin D_2_ administration than after vitamin D_3_ administration. Because there were no differences in 1,25(OH)_2_D generated after administration of vitamin D_2_ or vitamin D_3_, it is likely that the more rapid disappearance of 25(OH)D after vitamin D_2_ administration than after vitamin D_3_ administration is due to a greater rate of catabolism of 25(OH)D_2_, either by CYP24 [baseline levels of 24,25(OH)_2_D and 24,25(OH)_2_D:25(OH)D ratios were greater in the vitamin D_2_ group than in the vitamin D_3_ group] or by CYP3A4. Although it is possible that the greater apparent rate of catabolism of 25(OH)D_2_ is due to intrinsic differences between this metabolite and 25(OH)D_3_ as enzyme substrates, we cannot exclude the contrasting possibility that vitamin D clearance pathway(s) were more active in the children who received vitamin D_2_, particularly because clinical rickets seemed more severe in the vitamin D_2_ group than in the vitamin D_3_ group.

Elevated 1,25(OH)_2_D concentrations prior to treatment and a marked increase in 1,25(OH)_2_D concentrations in response to vitamin D reliably distinguished children with putative dietary calcium-deficiency rickets from healthy children and are consistent with functional vitamin D deficiency and/or secondary hyperparathyroidism in children with calcium-deficiency rickets. We confirmed that this increase in 1,25(OH)_2_D occurred in response to both vitamin D_3_ and vitamin D_2_ administration. A lack of response in 1,25(OH)_2_D concentrations to the administration of vitamin D or 25(OH)D has been used to define vitamin D sufficiency(10); thus, by this definition, the rachitic children would be defined as having vitamin D insufficiency. In children with dietary calcium-deficiency rickets, high production rates of 1,25(OH)_2_D probably represent an exaggerated parathyroid hormone (PTH)–mediated stimulation of 1α-hydroxylase owing to inadequate calcium intake. Of note, the increase in 1,25(OH)_2_D concentration after vitamin D administration in Nigerian children with rickets does not produce a corresponding increase in fractional calcium absorption(20) probably because intestinal calcium absorption (72%) is already maximal. We propose that primary calcium deficiency in Nigerian children induces an increase in 1α-hydroxylase activity that, combined with increased activity of degradative pathways (eg, CYP24), results in a state of relative vitamin D deficiency.(12) Based on a growing body of evidence, it is likely that reduced serum levels of 25(OH)D have an independent and adverse effect on bone and mineral metabolism in these children, leading both to increased secretion of PTH(23,24) and impaired bone mineralization.(25)

Despite their greater baseline 1,25(OH)_2_D concentrations, rachitic children had lower baseline 24,25(OH)_2_D concentrations than control children. 1,25(OH)_2_D is known to induce the expression of CYP24A1, the 24-hydroxylase enzyme responsible for catabolism of vitamin D.(13) We had expected to find greater 24,25(OH)_2_D concentrations or increased ratios of 24,25(OH)_2_D_3_:25(OH)D in children with rickets than in control children, consistent with induction of CYP24 by elevated concentrations of 1,25(OH)_2_D.(14) In fact, we found the opposite: Children with rickets had lower ratios of 24,25(OH)_2_D:25(OH)D than control subjects, similar to the changes in 24,25(OH)_2_D that occur in children with nutritional rickets.(1,26) These observations do not necessarily refute the notion that low baseline levels of 25(OH)D in the rachitic children are due to increased degradation because 24,25(OH)_2_D and 25(OH)D may be processed to other vitamin D metabolites or eliminated directly. However, we cannot exclude insufficient supply of vitamin D (eg, inadequate sunlight exposure) as a contributing factor. Careful analysis of the clearance rate of 25(OH)D would be necessary to confirm the increased rate of catabolism of 25(OH)D by CYP24 or other pathways (eg, CYP3A4).(27)

Some of our findings are consistent with more rapid metabolism of vitamin D_2_ than vitamin D_3_. One day following vitamin D administration, the concentration of serum vitamin D_2_ was lower in control children who received vitamin D_2_ than the vitamin D_3_ concentration in control children who received vitamin D_3_. At the same time, the 25(OH)D concentration rose more rapidly (*p* = .05 for difference in day 1 increment) and was followed by an earlier decline in children with rickets who received vitamin D_2_ compared with rachitic children who received vitamin D_3_. In both children with rickets and control children, the concentrations of 24,25(OH)_2_D rose more rapidly and declined earlier after vitamin D_2_ administration than after vitamin D_3_ administration. More rapid metabolism of vitamin D_2_ than vitamin D_3_ could reflect lower affinity for vitamin D–binding protein, increased affinity for the 24-hydroxylase CYP24 enzyme, and/or increased affinity for the 1α-hydroxylase CYP27B1.

Our previous study of the response to vitamin D_2_ in Nigerian children with rickets was limited by the absence of a control group without rickets.(12) This study thus offers several advantages over our previous work. For example, the findings of this study are strengthened by inclusion of control groups of healthy children, and we were able to compare the responses to vitamin D_2_ and vitamin D_3_. However, this report also has several limitations. The children with rickets who received vitamin D_2_ were a historical comparison group that was studied with an identical protocol. Consequently, their baseline characteristics differed from rachitic children who received vitamin D_3_, and the laboratory methods for determining vitamin D metabolites were different. However, we were able to demonstrate on stored sera the comparability of laboratory methods. We elected to use the historical vitamin D_2_ group of children with rickets because randomly assigning the group with rickets in this study to receive either vitamin D_2_ or vitamin D_3_ would have resulted in only half the number of children with rickets in each group, which would have resulted in reduced power in an already limited sample size. By using the same methodology and the same laboratory, we could compare the results directly without having to repeat the vitamin D_2_ challenge in a group of children with rickets. An additional shortcoming of this report includes the fact that the rachitic children who received vitamin D_2_ appeared to be more severely affected than the children who received vitamin D_3_. Finally, the lack of information on PTH concentrations in any of the groups of children makes our interpretation on the possible differences in factors driving CYP27B1 speculative.

We conclude that in the short term, vitamins D_2_ and D_3_ similarly increase 25(OH)D concentrations in rachitic and healthy children. However, vitamin D_2_ may be metabolized more rapidly than vitamin D_3_, thus supporting previous recommendations that cholecalciferol be considered the vitamin D of choice for the treatment of rickets. A marked increase in 1,25(OH)_2_D concentration in response to administration of vitamin D distinguishes children with putative dietary calcium-deficiency rickets from healthy children, thus indicating that vitamin D plus calcium should be used together in the treatment of nutritional rickets whether vitamin D or calcium deficiency is the primary etiology.
